# Low-dose β-carotene Supplementation and Deworming Improve Serum Vitamin A and β-carotene Concentrations in Preschool Children of Bangladesh

**DOI:** 10.3329/jhpn.v28i3.5549

**Published:** 2010-06

**Authors:** Rashidul Haque, Tanvir Ahmed, M.A. Wahed, Dinesh Mondal, A.S.M. Hamidur Rahman, M. John Albert

**Affiliations:** ^1^ ICDDR,B, GPO Box 128, Dhaka 1000, Bangladesh; ^2^ Department of Microbiology, Faculty of Medicine, Kuwait University, PO Box 24923, Safat 13110, Kuwait

**Keywords:** Albendazole, Antihelminthics, Ascariasis, *Ascaris lumbricoides*, β-carotene, Children, Deworming, Impact studies, Retinol, Vitamin A deficiency, Bangladesh

## Abstract

Despite the national vitamin A and antihelminthic prophylaxis programmes, both intestinal geo-helminths and subclinical vitamin A deficiency continue to be prevalent among children in developing countries. Studies on potential synergistic effects of vitamin A supplementation and deworming on retinol status have inconsistent results. The purpose of the present study was to investigate the impacts of low-dose β-carotene supplementation and antihelminthic therapy on serum retinol and β-carotene concentrations in preschool children of Bangladesh. Two hundred and forty-four children, known to be infected with *Ascaris lumbricoides*, were randomized into four treatment groups: I-IV. Group I and II received two oral doses of 400 mg of albendazole each, the first dose at baseline and the second dose after four months; Group III and IV received placebo in place of albendazole. In addition, Group I and III received 1.2 mg of β-carotene powder in capsule daily for six months, and Group II and IV received placebo in place of β-carotene. Serum retinol and β-carotene levels were measured before and after six months of the interventions. Serum retinol and β-carotene increased significantly in Group I where both antihelminthic therapy and daily β-carotene supplementation were given (p<0.05 and p<0.001 respectively). Antihelminthic therapy alone only improved serum β-carotene concentration (p<0.0001). Low-dose β-carotene supplementation, along with an antihelminthic therapy, synergistically improved vitamin A status. This finding has public-health implications for improving vitamin A status of children in developing countries.

## INTRODUCTION

It is recognized that both clinical and subclinical vitamin A deficiencies (VAD) are a serious public-health problem, particularly in developing countries (1–6). Eradication of VAD has a positive impact on growth, morbidity, and mortality in children (7, 8). The World Health Organization (WHO) recommended strategies that have been implemented in developing countries for more than three decades to eradicate VAD. These include: (a) high-dose vitamin A capsule supplementation, (b) food fortification or diversification, and (c) nutritional awareness. Although the national vitamin A prophylaxis programmes have been implemented for a long time, VAD continues to be a major nutritional catastrophe of public-health significance in many countries (1, 9).

The efficacy of programmes to improve vitamin A nutriture may be reduced, however, in areas where soil-transmitted intestinal parasites are still common. An estimated 4.5 billion people of the world are at risk of intestinal parasites, and 1.2 billion might be infected with roundworms, such as *Ascaris lumbricoides* (10, 11). Intestinal parasites, particularly roundworms, impair the absorption of many nutrients, including vitamin A and, thus, may render improvements in dietary intake of vitamin A ineffective (12, 13). Further, it may be true that people suffer from such deficiencies solely because of the decreased availability of adequate micronutrients from the diet due to intestinal parasites; this problem has not been adequately studied.

Some researchers have found a relationship between indicators of micronutrient deficiency and infection due to *A. lumbricoides*. Serum vitamin A and carotenoid were significantly lower in *Ascaris*-infected children in India, Nepal, and Panama (14–17). In India, school children infected with *A. lumbricoides* had significantly impaired absorption of retinol compared to normal controls (18). In a subsequent study, vitamin A absorption improved significantly after deworming in 13 of 14 adults with ascariasis (13). The authors suggested deworming to be one of the means of improving vitamin A status (13). Results of several other studies showed that the rise of serum retinol occurred when the children were dewormed, along with other food-based interventions (19–21). Studies also documented that helminthic infections are important causes of iron-deficiency anaemia in Asia and sub-Saharan Africa (22–24) and deworming programmes can improve the iron status and prevent moderate-to-severe anaemia (25–28). Integrated health implications of vitamin A deficiency and of *A. lumbricoides* are well-documented; however, our understanding of their combined biological interactions is limited. The WHO recently recommended the use of antiheliminthic drug treatment to control ascariasis and megadoses of vitamin A supplements to reduce vitamin A deficiency (29). In accordance with other developing counties, the Government of Bangladesh has also adopted the antiheliminthic and vitamin A supplementation programmes on the national immunization days (30); however, their potential synergistic effects on health, particularly serum retinol levels to monitor the subclinical vitamin A deficiency, have not been well-documented. Studies investigating this have provided inconsistent findings for whether antihelminthic therapy enhances potential benefits of higher absorption of vitamin A or β-carotene from foods, or vitamin A from supplements (19, 20, 31, 32). In Bangladesh, both ascariasis and vitamin A deficiency are prevalent in children (33, 34). The major objective of this study was to examine the separate and combined impacts of low-dose β-carotene supplementation and antihelminthic therapy on serum retinol and β-carotene concentrations in preschool children. The daily low-dose β-carotene supplementation, as it is done in this study, is not seen as a sustainable intervention but as a proxy for an improved dietary intake.

## MATERIALS AND METHODS

### Subjects

The study was conducted among children of a slum community in Mirpur, Dhaka, Bangladesh. Mirpur is one of the 14 thanas (subdistricts) of Dhaka city with a population of about one million in an area of 59 sq km. The population of Mirpur is stable with low socioeconomic conditions which is similar to other parts of Dhaka city. The average income is Tk 4,200 (about US$ 68) per month per family. Twenty-five percent of fathers and 15% of mothers have more than five years of formal education.

Preschool children, aged 24–60 months, were enrolled from Mirpur. A list of all eligible children was prepared from the area. In total, 248 children of both sexes were selected from among the children with infection due to *A. lumbricoides*. After informed consent was obtained from the head of the household, trained Health Assistants, using a standardized and pretested questionnaire regarding the health of the children, interviewed the mothers of these children at their houses. The inclusion criteria for the study children were: (a) apparently healthy without a history of chronic illness; (b) without hookworm infection, and (c) willing to take daily β-carotene capsule and two doses of albendazole during the study.

The Research Review Committee and the Ethical Review Committee of ICDDR,B approved the study.

### Stool examinations

Stool samples were collected in wide-mouthed plastic bottles for microscopic examinations. All stool samples were examined by direct microscopy using a wet smear. Trophozoites and cysts of all protozoan parasites, including *Giardia lamblia*, were looked for*.* Stool samples were examined using a formalin-ether concentration technique (35) at the Parasitology Laboratory of ICDDR,B. About one g of stool was transferred to a falcon-tube containing 10% formalin-saline, and the sample was processed according to the established method. After concentrating the sample in the tube, a few drops of normal saline were added, and the total drops in the tube were counted. One drop from the tube was taken onto a glass-slide and examined under a microscope. The eggs in the sample were counted and expressed as eggs per g (epg) of stool to assess the intensity of helminthic infection. Eggs of *A. lumbricoides, Trichuris trichiura*, and hookworm were counted and expressed as epg. *Ascaris*-associated infection was a prerequisite for enrollment into the study, and children who had an egg count of ≥1,000 epg for *A. lumbricoides* were invited to participate in the study.

### Physical examinations and anthropometric measurements

A physician performed a complete physical examination of the children. The examination included information on immediate past and current childhood illnesses and anthropometric evaluation. Heights and weights of the children were assessed using standard procedures of the WHO (36). Seventy-five children with severe malnutrition, clinical vitamin A deficiency (as indicated by corneal involvement), chronic diseases, or persistent diarrhoea were referred to the Children's Hospital, Dhaka or ICDDR,B and were excluded from the study.

### Treatment regimens

Children, known to be infected with *Ascaris*, were randomized into four different treatment regimens (Group I-IV, Table 1). Treatments were oral doses of 400 mg of albendazole and daily 1.2 mg of β-carotene powder in capsules. Placebo forms for both albendazole tablets and β-carotene capsules were of identical size, shape, and colour. Albendazole tablets and β-carotene capsules were obtained from the Eskayef Bangladesh Ltd., Bangladesh and Tishcon Corporation, USA respectively. Group I and II received two oral doses of 400 mg of albendazole—one at baseline and another after four months—while Group III and IV received placebo tablets at baseline and after four months. Group I and III received daily β-carotene capsules while Group II and IV received placebo daily for six months. The Health Assistants administered albendazole tablets or placebo to the children while β-carotene or placebo capsules were given to the mothers for daily administration to the children. The first time, the Health Assistants administered β-carotene capsules in presence of the mothers. Whole capsules were either given or were opened and the contents poured into the child's mouth. The Health Assistants visited the households twice a week to monitor compliance in taking β-carotene capsules and to replenish the supply.

**Table 1.** Characteristics of study children infected with *A. lumbricoides* at baseline*

Parameter	Treatment group			
	Group I (n=52)	Group II (n=55)	Group III (n=58)	Group IV (n=56)
	Albendazole+β-carotene	Albendazole+ placebo	β-carotene+ placebo	Placebo+ placebo
Age (months)	45.9±1.5	43.9±1.5	46.2±1.4	43.8±1.3
Gender, % boys	52	62	57	48
Weight (kg)	12.2±0.3	12.3±0.3	12.4±0.3	12.4±0.3
Height (cm)	90.9±1.1	90.9±1.2	91.4±1.0	91.6±1.3
Height-for-age (%)†	90.4±0.9	91.7±0.9	90.6±0.9	91.9±1.0
Weight-for-age (%)	76.3±1.4	78.4±1.5	78.1±1.6	79.2±1.5
Weight-for-height (%)	90.6±0.8	87.7±3.5	92.6±1.3	91.5±0.9
*Ascaris lumbricoides* (egg)‡	3853±340	4923±551	4853±473	4689±426
*Trichiura* co-infection (%)	96.1	96.3	98.2	96.4

*Mean±SEM;

†Values are in relation to the World Health Organization standards (28);

‡Eggs per g of stool sample;

SEM=Standard error of the mean

### Randomization and sample-size

Block randomization was used for recruiting children in the treatment group and placebo (control) groups. In calculation of the sample-size, the level of significance (α) was 0.05 two-sided, and the power (β) was 0.90. The selection of differences between group means (β) was taken 30% in serum retinol and β-carotene level of every intervention group compared to the placebo (control) group. Using the same α and β and assuming a coeffiecient of variation for growth of 40% in this age-group, a sample-size of 37 per group is required. Taking into account the design effect and attrition, the sample-size increased to 51 per group, or total 204 children.

### Serum β-carotene and retinol determination

After antihelminthic therapy was given and before β-carotene supplementation, venous blood samples were collected from the children and were transported to the laboratory in cool boxes to protect from heat and light. A similar procedure was used after six months when low-dose β-carotene supplementation was completed. Blood was centrifuged, and serum was frozen at -20°C until analyses of retinol and β-carotene by high-pressure liquid chromatography (HPLC) (37–39) at the Nutritional Biochemistry Laboratory of ICDDR,B.

### Data management and analysis

The study employed a factorial design with deworming of two groups and daily β-carotene supplementation in the other two groups. Data were also analyzed according to factorial design. All data collected were computer-coded and analyzed with the SPSS software (version 7.5) (SPSS Inc, Chicago, IL, USA). All results were expressed as mean±SEM. Analysis of variance (ANOVA) was performed on the observed changes in serum retinol and β-carotene concentration according to the design of the study. To compare the baseline and follow-up values within the treatment groups, a paired *t*-test was used. Comparisons among means of different variables were made using *t*-test. Significance was defined as p≤0.05.

Control of potential confounding factors was not necessary because all children who had been identified as infected with *A. lumbricoides* before intervention and who were willing to be in the study were randomly assigned to the four treatment groups as the study began. Thus, the probable effects of the confounding factors on the results were included in the probability statements about statistical significance.

## RESULTS

Of the 248 children recruited, complete data were available from 221 (90%) after six months. Twenty-three children did not complete the trial for various reasons, including migration, refusal to take supplements, etc. In total, 858 children were examined for *A. lumbricoides*-associated infection at baseline. Seventy-eight percent of the children were positive for infection due to *A. lumbricoides*, and about two-thirds of them had an egg count of <1,000 epg for *A. lumbricoides*. The data on epg revealed that the children recruited had a wide range of intensity of infection. The median epg for *A. lumbricoides* was 4,593 (range 1,000–21,964 epg). According to the WHO criteria (35, 40), 67% of the children had light infection (<4,999 epg), 33% had moderate infection (5,000–49,999 epg), and none had heavy infection (≥50,000 epg) with *A. lumbricoides.* The intensity of *A. lumbricoides*-associated infection as measured by egg counts was similar between the treatment groups (Table 2). Ninety-six percent of the children were co-infected with *T. trichiura;* 56% of these had light co-infections with *T. trichiura* (1–999 epg) according to the WHO criteria. Infection due to hookworm was rare (<1%) in these young children. There were no significant differences (p>0.05) among the four groups of children at baseline in terms of age, weight, height, and nutritional status. There were similar numbers of boys and girls (Table 1). Both height and weight deficits compared to the WHO standards indicated that the study children were marginally malnourished.

**Table 2.** Drug efficacy and changes in helminthic infections in stools of children*

Helminth	Treatment group			
	Group I (n=52)	Group II (n=55)	Group III (n=58)	Group IV (n=56)
	Albendazole+ β-carotene	Albendazole+ placebo	β-carotene+ placebo	Placebo+ placebo
Baseline stool examination				
*Ascaris lumbricoides* (epg)	3854±340†	4923±551	4853±474	4689±426
*Trichuris trichiura* (epg)	1082±159	1311±188	1310±136	1571±194
Final stool examination				
*Ascaris lumbricoides* (epg)	1±1	19±12	4332±657	4525±738
*Trichuris trichiura* (epg)	83±41	205±63	880±125	1052±201
Children with eggs at final examination				
*Ascaris lumbricoides*	1	3	52	45
*Trichuris trichiura*	10	18	54	49
Cure rate (%)‡				
*Ascaris lumbricoides*	98.1	94.5	10.3	19.6
*Trichuris trichiura*	80.8	67.3	6.9	12.5

*The first stool examination was done at the time of initial subject recruitment, and the final examination was done after 4 months before the second antihelminthic drug and placebo were given;

†Mean±SEM;

‡Calculated as the percentage of those with no *A. lumbricoides* and *T. trichiura* eggs observed in the stool compared to the total number of children examined in the group;

SEM=Standard error of the mean

The efficacy of antihelminthic drug treatment is shown in Table 2. The results showed that the drug was highly effective in eliminating infection due to *A. lumbricoides* but only 67–81% of *T. trichiura* were cured in children of Group I and II. The children of Group III and IV who received placebo remained infected with both *A. lumbricoides* and *T. trichiura*. Of 107 children in Group I and II, four remained infected with *A. lumbricoides* at final stool examination. Of 114 children in Group III and IV, 97 remained infected with *A. lumbricoides* (Table 2). There was no correlation between *A. lumbricoides* egg counts at baseline and serum retinol and β-carotene concentrations at baseline. When the data were stratified according to the intensity of *A. lumbricoides*-associated infection based on stool egg counts, ANOVA showed that there were no significant differences between the lightly (epg <5,000)- and heavily (epg ≥5000)-infected children in all the groups on serum retinol and β-carotene at baseline and after six months (data not shown).

The compliance rate of taking β-carotene supplementation among the study subjects was >95%. The serum β-carotene and retinol levels at baseline and after six months in the four groups of children are shown in the figure. The mean serum β-carotene and retinol concentrations in all the groups were 0.16 μmol/L and 0.66 μmol/L respectively. There were no significant differences (p>0.05) among the groups in baseline values of serum retinol and β-carotene. Within the treatment groups, the serum β-carotene concentration increased significantly after six months in all the groups, except the control group (Group IV) but a significant increase of serum retinol occurred only in Group I and Group III (Fig.).

**Fig.**
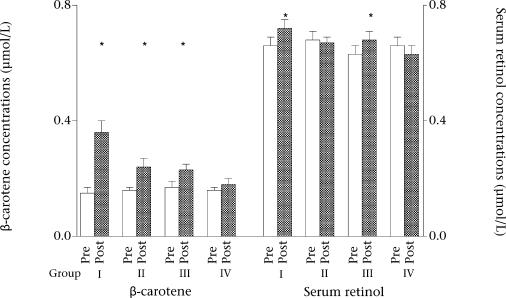
Effect of antihelminthic therapy with albendazole and daily low-dose β-carotene supplementation on serum retinol and β-carotene levels

*Indicates statistical significance at p<0.05 between baseline and after 6 months of β-carotene supplementation using the Wilcoxon signed rank test Group I, II, III, and IV represent albendazole+ β-carotene, albendazole+placebo, β-carotene+placebo and placebo+placebo recipients respectively. Data are mean±SEM of serum retinol and β-carotene levels before (pre) and after (post) supplementation of β-carotene in Bangladeshi children;
SEM=Standard error of the mean

In total, 127 (57.5%) children were vitamin A-deficient (serum retinol of ≤0.7 μmol/L), of which 15 (6.8%) were severely deficient (<0.35 μmol/L). Over 95% of the children (211 of 221) had serum β-carotene of <0.35 μmol/L. The vitamin A-deficient children were equally distributed into four groups (data not shown).

The figure shows the changes of serum β-carotene and retinol after six months. The ANOVA revealed that both antihelminthic therapy and β-carotene supplementation significantly improved serum β-carotene (Fig.), and the combined effect was synergistic. The independent effects were 0.08 and 0.06 μmol/L for deworming and β-carotene supplementation respectively, which would sum to 0.14 μmol/L if their combined effects were additive. In fact, their combined effect was 0.21 μmol/L, which was greater than the additive effect. No such synergistic effect was seen for serum retinol (Fig.). However, β-carotene-supplemented and dewormed children improved in serum retinol significantly compared to the control group (Group IV). Children who were supplemented only with β-carotene (Group III) also improved their serum retinol level compared to the control group.

## DISCUSSION

The purpose of the study was to determine the effects of daily supplementation with low-dose β-carotene and/or antihelminthic therapy with albendazole on the vitamin A status of preschool children of Bangladesh, who were generally free of clinical manifestations of vitamin A deficiency but were known to be infected with *A. lumbricoides*. The goal of the trial was to establish whether one intervention is more effective than the other or whether a combination of these interventions is required to improve the vitamin A status in these children.

Both serum β-carotene and retinol concentrations were used as markers of vitamin A status of various treatment groups at baseline and after six months of the intervention. About 57% of the children had marginal vitamin A deficiency (serum retinol of <0.7 μmol/L). The children were, thus, appropriately selected to study the effects of both deworming and low-dose β-carotene supplementation on vitamin A status. A recent study has shown that the serum retinol concentrations were significantly different when the data were stratified according to the intensity of *A. lumbricoides*-associated infection (19). In the present study, we have not observed any significant differences of serum retinol and β-carotene concentration when the data were stratified according to the intensity of infection. The reason for this could be that we recruited only those children who had an epg of at least 1,000 or more for *A. lumbricoides.*

Results of studies also showed that deworming, along with food-based interventions, improved the vitamin A status of Filipino and Indonesian children (19, 21). However, the designs of the studies did not allow to answer the question of whether deworming in the absence of added fat or dietary β-carotene would improve serum retinol. Our results suggest that low-dose β-carotene supplementation is effective in improving the vitamin A status of children while antihelminthic therapy has a synergistic effect when given with low doses of β-carotene supplementation. Treatment only with antihelminthic therapy has an effect on serum β-carotene concentration but not on serum retinol. This may be due to difficulties in the conversion of the dietary sources of β-carotene to retinol in these children. This conversion occurs in the gut's brush border cells before it is absorbed. *Ascaris*-associated infection affects functions of the cells of the intestinal mucosa (13, 18, 41). The effectiveness of dietary vitamin A precursors may be influenced by several dietary and environmental factors. One of these factors may be the plant structure in which β-carotene is found (19, 42, 43). Another study reported that β-carotene was poorly absorbed from dark-green leafy vegetables but that absorption from a pharmaceutical source was good (44, 45). The fact that deworming improved the serum β-carotene level in this study is of major public-health significance since carotenoid-rich food provides 80% of vitamin A consumed by this population. In many parts of the world, where poor sanitation, high prevalence of *A. lumbricoides*, and childhood malnutrition prevail, especially vitamin A deficiency, there is reason to believe that *Ascaris*-associated infection can contribute to vitamin A deficiency (9, 14–17, 28). Thus, effective control of intestinal helminths can be a useful nutritional intervention to improve the vitamin A status of children, especially those children whose consumption of dietary fat and vitamin A is marginal.

Marinho *et al.* also found a good response to the retinol supplementation only in children who were treated for intestinal parasitic infection (31). In the present study, we have demonstrated that, in preschool children with subclinical vitamin A deficiency, who are also infected with intestinal parasites, low-dose β-carotene supplementation with antihelminthic therapy synergistically improved the vitamin A status. Efforts have been made to use local food sources of vitamin A precursors from green-leafy vegetables and fruits to improve the vitamin A status. The results of our study indicate that the national programmes for deworming and vitamin A supplementation prophylaxis, along with other food-based interventions that will provide precursors of vitamin A, may be useful in improving the vitamin A status of preschool children in countries where both vitamin A deficiency and high prevalence of intestinal helminths exist. However, this presumption now needs to be tested because our study used synthetic β-carotene.

## ACKNOWLEDGEMENTS

The study was funded by the Thrasher Research Fund, USA (Grant No. 02812-2). Antihelminthic drug ®Alben (albendazole) was provided by the Eskayef Bangladesh Ltd. ICDDR,B acknowledges with gratitude the commitment of the Thrasher Research Fund and Eskayef to the Centre's research efforts.

The authors also thank the families and children of Mirpur study area for their participation in the study.
